# Impact of the COVID-19 pandemic on the outcomes of colorectal cancer surgical patients treated at a public hospital in Southern Brazil

**DOI:** 10.3389/fsurg.2025.1591216

**Published:** 2025-07-15

**Authors:** Thiago Lucas Bastos de Melo Moszkowicz, Tierre Aguiar Gonçales, Mariana Severo Debastiani, Gabriela Klein Herwig, Laura Martin Manfroi, Carolina de Moura Marolli, Rafael José Vargas Alves, Claudia Giuliano Bica

**Affiliations:** ^1^ Pathology Post Graduation Program from Federal University of Health Sciences (Universidade Federal de Ciências da Saude de Porto Alegre), Porto Alegre, Brazil; ^2^ Medicine Graduation Program at Federal University of Health Sciences (Universidade Federal de Ciências da Saude de Porto Alegre), Porto Alegre, Brazil; ^3^ Biomedicine Graduation Program at Federal University of Health Sciences (Universidade Federal de Ciências da Saude de Porto Alegre), Porto Alegre, Brazil; ^4^ Internal Medicine Department of Federal University of Health Sciences (Universidade Federal de Ciências da Saude de Porto Alegre), Porto Alegre, Brazil; ^5^ Saint Rita Hospital of Irmandade Santa Casa de Misericórdia de Porto Alegre (Brotherhood of the Holy House of Mercy of Porto Alegre), Porto Alegre, Brazil; ^6^ Department of Basic Health Sciences of Federal University of Health Sciences (Universidade Federal de Ciências da Saude de Porto Alegre), Porto Alegre, Brazil

**Keywords:** colorectal cancer, COVID-19, SARSCOV-2, surgery, pandemic, oncological surgery, public health system

## Abstract

**Purpose:**

The pandemic has had a clear impact on surgical procedures, leading to a significant decline in volume due to the postponement of non-emergency operations. This has permitted healthcare providers to reallocate personnel and resources to address the ongoing coronavirus pandemic. The present study aims to assess the impact of the COVID-19 pandemic on mortality and the evolution of the clinical condition of colorectal tumors in surgical patients in a hospital unit of the Brazilian public health system.

**Methods:**

This is a cohort study evaluating 263 adult patients with colorectal tumors who underwent surgery between January 2019 and March 2020 (pre-pandemic period) and between March 2020 and April 2021 (pandemic period) in the Unified Health System (SUS) in a tertiary hospital in southern Brazil. The first follow-up was carried out 30 days after surgery and the second at the end of the participants' hospitalization period. Primary Outcome(s) and Measure(s): The primary outcome was mortality up to 30 days after surgery. Secondary outcomes related to coronavirus infection included length of hospital stay, intensive care unit admission, overall mortality, emergency or palliative surgery, histopathological variables and advanced-stage tumors. Logistic regression was used to determine the potential relationship between the pandemic period and the outcomes of advanced cancer, emergency surgery, and 30-day mortality.

**Results:**

The study included 263 patients; 145 (55.1%) underwent surgery during the pandemic period and 118 (44.9%) in the pre-pandemic period. Coronavirus infection was associated with increased 30-day mortality, death, length of stay, and admission to the intensive care unit. Moreover, logistic regression confirmed that during the pandemic period, emergency surgeries increased but did not affect advanced cancer progression or 30-day mortality in cancer patients.

**Conclusions:**

The pandemic period did not significantly influence overall mortality or 30-day mortality, nor did it affect the incidence of advanced cancer. These findings underscore the critical importance of maintaining surgical operations during pandemic periods.

## 1 Introduction

The advent of the novel coronavirus disease pandemic precipitated an urgent need to rapidly reorganize health systems at the global and national levels (1–6). Even at the onset of the pandemic, when knowledge about the virus was limited, non-urgent procedures needed to be reorganized. In this context, it is also imperative to acknowledge the prevailing concern among patients seeking healthcare services for non-coronavirus-related issues, as well as those who were apprehensive about contracting the virus. This apprehension has had a substantial impact on a significant portion of the population (7).

The Coronavirus Disease 2019 (COVID-19) pandemic has placed additional stress on cancer survivors and healthcare systems. As the pandemic continues to substantially impact the world, it is critical to focus attention on the healthcare needs of cancer survivors (8). Management of colorectal cancer (CRC), the third most common cancer worldwide, was markedly affected by the pandemic (9). Colonoscopies and colorectal cancer screening tests were reduced substantially in 2020 (10). At the same time, oncology and surgery societies altered treatment guidelines, favoring the postponement of surgery. According to the Brazilian Society of Pathology and Oncological Surgery, over 50,000 Brazilians had their cancer diagnoses affected by the pandemic by the first half of 2020, the first year of the COVID-19 pandemic (11).

It is an indisputable fact that patients with cancer patients experience significant biopsychosocial changes. The experience of the 2020 novel coronavirus (SARS-CoV-2) pandemic negatively impacted the quality of life of cancer patients. This is due to several factors, including their heightened susceptibility to infection, immunosuppression, the probable delay in diagnosis and treatment, and the routine transformation of physical activities and nutritional intake (12). Given their higher median age, CRC patients, in particular, are at an increased risk for severe symptoms and complications from infection, especially when immunosuppressed (13–16).

A multitude of studies have demonstrated that numerous nations have encountered challenges in providing adequate cancer care in the context of the pandemic (1, 6). A cohort study in Italy demonstrated a clear link between the SARS-CoV-2 pandemic and an increased risk of patients with colorectal cancer being diagnosed with more advanced stages of the disease (2). This suggests a potential reduction in the survival rate of these patients.

In summary, the pandemic period has required the health system to adapt in order to meet the demands of patients infected with the SARS-CoV-2 virus and minimize nosocomial contamination, especially for patients with CRC. Therefore, the present study will assess the impact of the COVID-19 pandemic on the care of CRC surgical patients treated by the Sistema Único de Saúde (SUS) in a tertiary hospital in southern Brazil in 2020 (during the pandemic) compared to 2019 (before the pandemic).

## 2 Methods

### 2.1 Study design and participants

This retrospective cohort study involves cancer patients admitted to a referral hospital in southern Brazil for surgery between January 2019 and April 2021. The hospital's Ethics Committee approved the study under CAAE number 51129321.2.0000.5335 and registered on the Brazil Platform under number 5.061.942.

The inclusion criteria were clear: data from oncological surgeries (open or laparoscopic abdominoperineal amputation of the rectum; partial and total colectomy; colostomy; total proctocolectomy; and open or laparoscopic abdominal rectosigmoidectomy) in adult patients with colorectal cancer, performed by the Unified Health System (SUS) from January 2019 to April 2021. Data from private and health insurance surgeries, pediatric surgeries, and cases that lacked complete information were excluded from this study.

The variables collected were identified using institutional electronic medical records (TASYR). A team of researchers entered the data into a REDCap database (Research Electronic Data Capture; Vanderbilt University), respecting the confidentiality of the information. The checklist was based on the verification items following the STROBE methodology. The study followed the Declaration of Helsinki and the General Data Protection Regulation (GDPR), guaranteeing data confidentiality and patient privacy.

We analyzed the following variables: age, hospitalization data (date, need for ICU, the outcome with discharge or death, date of outcome), surgery data (date, ASA, urgent or palliative status, mortality within 30 days of surgery), and neoplasm data (target neoplasm site, pathology information, number of positive lymph nodes and distant metastasis [MET], and COVID-19 status for patients treated during the pandemic (PCR test for SARS-CoV2, test date, and result). Tumors were staged from I to IV according to the 8th edition of the American Joint Committee on Cancer (6).

The outcome of the advanced cancer stage was assessed based on the following criteria: patients who underwent palliative surgery and/or had an unresectable tumor and AJCC classification of advanced tumor from stage IIB.

The primary outcome of this study was mortality at 30 days after surgery in the pre-pandemic and pandemic cohorts. Secondary outcomes included length of stay, need for ICU, death, and follow-up 30 days after hospital discharge, tumor aggressiveness, advanced cancer, need for emergency or palliative surgery, and unresectable neoplasia.

The pre-pandemic period was from January 2019 to March 10, 2020. The period related to the SARS-CoV2 pandemic began on March 11, 2020, when the WHO declared a global pandemic (17), and ended on April 1, 2021, when the first vaccinations were administered in the region where the hospital is located, in the state of Rio Grande do Sul.

All researchers involved in data collection were trained and double-checked to avoid bias. The study used a stratified sample based on the different types of neoplasms that required surgical intervention during the study period. The variables were analyzed according to this stratification and whether the period was pandemic or not.

### 2.2 Statistical analysis

Continuous numerical variables were expressed as mean ± standard deviation (SD), while nominal categorical variables were expressed as number (absolute frequency) and percentage (relative frequency). The results were subjected to a homogeneity test and normality test to determine the statistical analysis to be carried out.

We performed a Shapiro–Wilk test on the only continuous variable analyzed in our study, “days in hospital,” for both the primary analysis and the COVID subgroup analysis. The variables were not normally distributed for the pandemic and pre-pandemic periods (pandemic: *p* < 2.2 × 10^−16^; pre-pandemic: *p* < 4.5 × 10^−11^). Similarly, “days in hospital” was not normally distributed for the positive vs. negative subgroups (positive: *p* = 1.06 × 10^−5^; negative: *p* = 3.96 × 10^−5^). Therefore, Student's *t*-tests or Mann–Whitney tests were used to compare data between the two groups (surgical patients in the pre-pandemic and pandemic periods) for quantitative variables, and chi-square or Fisher's exact tests were used for qualitative (nominal) variables. Logistic regression was used to assess a possible relationship between the outcomes and the variables age, gender, and pandemic period.

For the analysis of complication severity, we classified each patient according to the Clavien-Dindo classification (18). We excluded surgical reinterventions to avoid duplication of cases and included only the first intervention per patient. We analyzed the data using three categories (I-II, III, and IV) for the pandemic vs. pre-pandemic subgroup. However, due to the small number of Grade III cases, we performed a simplified two-category analysis (Grade I-II vs. Grade III-IV) to improve robustness. We used the same simplified, two-category approach for the subgroup of patients who tested positive for or negative for SARS-CoV-2.

The significance level was set at 0.05, and *P* < 0.05 was considered significant. The confidence interval was set at 95%. R Studio was the program used for the statistical calculations.

## 3 Results

**Figure 1:**
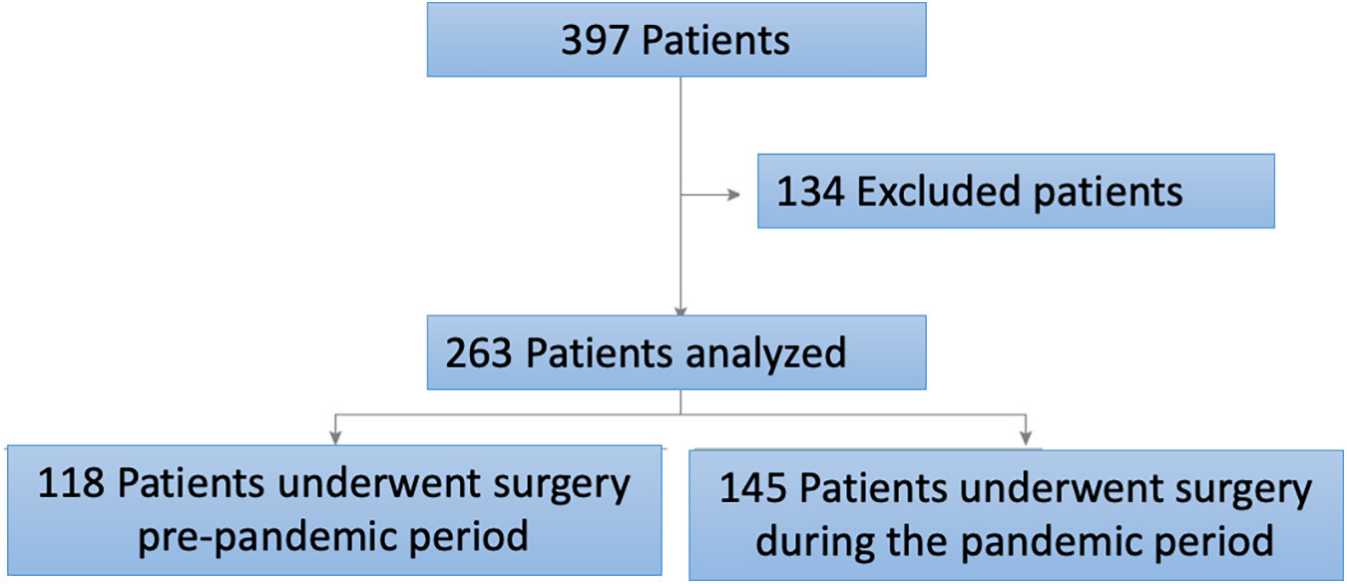
Flowchart of the study during the pre-pandemic period vs. the pandemic period.

This study included 263 patients, as shown in Table 1 and Figure 1. The study analyzed 145 cancer patients (55.1%) who underwent surgeries during the SARS-CoV-2 pandemic and 118 (44.9%) who underwent surgeries in the pre-pandemic period.

**Table 1:** Characteristics of the population sample by period of surgery.

Variables	Pandemic period, *N* = 145^a^	Pre-pandemic period, *N* = 118^a^	Total^a^, *N* = 263	*p*-value^b^
Female	71 (48.96%)	67 (56.77%)	138 (52.47%)	0.20
Age				0.58
<60 years	44 (30.34%)	40 (33.89%)	84 (31.93%)	0.53
60–69 years	45 (31.03%)	35 (29.66%)	80 (30.41%)	0.81
70–79 years	44 (30.34%)	29 (24.57%)	73 (27.75%)	0.29
≥80 years	12 (8.27%)	14 (11.86%)	26 (9.88%)	0.32
Death outcome	15 (10.34%)	13 (11.01%)	28 (10.64%)	0.86
Days in hospital	28.35 (35.16)	33.69 (26.97)	30.95 (30.67)	**0** **.** **086**
Admission to the Intensive Care Unit	53 (36.55%)	61 (51.69%)	114 (43.34%)	**0** **.** **011** *
30-day mortality	14 (9.65%)	9 (7.62%)	23 (8.74%)	0.20
Emergency surgery	42 (28.96%)	19 (16.10%)	62 (23.57%)	**0** **.** **014** *
Paliative surgery	10 (6.89%)	10 (8.47%)	20 (7.60%)	0.63
ASA				0.3
1	7 (4.82%)	2 (1.69%)	9 (3.42%)	0.14
2	80 (55.17%)	78 (66.10%)	158 (60.07%)	0.072
3	51 (35.17%)	36 (30.50%)	87 (33.07%)	0.42
4	3 (2.06%)	2 (1.69%)	5 (1.90%)	>0.9

Bold values indicate results considered relevant for interpretation (including statistically significant results and trends).

^a^ n (%); Mean (SD).

^b^ Pearson's Chi-squared test; Fisher's exact test; Wilcoxon rank sum test.

* Statistical significance (*p* < 0.05).

### 3.1 Severity of complications: statistical analysis

In the two-category analysis, severe complications (grades III and IV) occurred significantly less frequently during the pandemic (36.6%) than in the pre-pandemic period (52.3%) (Fisher's exact test, *p* = 0.0152). We also analyzed complication severity in the subgroup of patients who were positive for SARS-CoV-2 vs. those who were negative. No statistically significant difference in complication severity was observed between the two groups (Fisher's exact test, *p* = 0.0647). However, a trend toward more severe complications in the positive group was observed (50% vs. 24%).

As seen in Table 1, 145 patients underwent surgery during the pandemic. This was associated with a higher rate of emergency surgery, a lower need for intensive care, and fewer days in the hospital compared to the pre-pandemic period (*n* = 118).

The data show a change in the pattern of emergency surgeries, length of hospital stays, and need for intensive care during the pandemic. However, 30-day mortality or death outcomes were not altered during the pre-pandemic period compared to the pandemic period (Table 1).

Regarding staging, according to the AJCC, the tumors showed no statistically significant difference between the pre-pandemic and pandemic periods, whether evaluated together or separately.

As shown in Table 2, there was no statistically significant difference in the number of cases between the two periods for T1 and T4 tumor stages. T2 and T3, on the other hand, showed a statistically significant difference between the pre-pandemic period and the pandemic period. This indicates that milder and more severe cases (T1 and T4) remained consistent during the pandemic period. However, among moderate cases (T2 and T3), there was an apparent increase in severity during the pandemic period.

**Table 2:** Histological characteristics of the sample by surgery period.

Variables	Pandemic period, *N* = 145^a^	Pre-pandemic, *N* = 118^a^	Total^a^, *N* = 263	*p*-value^b^
N				0.8
N0	71 (48.96%)	65 (55.08%)	136 (51.71%)	0.323
N1 (a-c)	37 (25.51%)	28 (23.72%)	65 (24.71%)	0.738
N2 (a-c)	24 (16.55%)	18 (15.25%)	42 (15.96%)	0.775
N3 (a-b)	0 (0%)	0 (0%)	0 (0%)	-
Metastasis	8 (5.51%)	5 (4.23%)	13 (4.94%)	0.644
AJCC Staging				0.424
I	21 (14.48%)	25 (21.18%)	46 (17.49%)	0.155
IIA	4 (2.75%)	26 (22.03%)	68 (25.85%)	0.202
IIB	2 (1.37%)	7 (5.93%)	9 (3.42%)	0.083
IIC	6 (4.13%)	5 (4.23%)	11 (4.18%)	>0.9
IIIA	6 (4.13%)	8 (6.77%)	14 (5.32%)	0.343
IIIB	37 (25.51%)	24 (20.33%)	61 (23.19%)	0.322
IIIC	14 (9.65%)	11 (9.32%)	25 (9.50%)	>0.9
IV	3 (2.06%)	2 (1.69%)	5 (1.90%)	>0.9
IVA	5 (3.44%)	4 (3.38%)	9 (3.42%)	>0.9
IVC	0 (0%)	1 (0.84%)	1 (0.38%)	0.449
Advanced cancer	81 (55.86%)	62 (52.54%)	143 (54.37%)	0.591
T				0.009
T1	13 (9%)	5 (4.2%)	18 (6.84%)	0.131
T2	14 (9.7%)	29 (24.6%)	43 (16.34%)	0.001
T3	90 (36.7%)	61 (51.7%)	151 (57.41%)	0.007
T4	28 (19.3%)	22 (18.6)	50 (19.01%)	0.891
Histological type				0.296
Adenocarcinoma	135 (93.1%)	113 (95.8%)	248 (94.3%)	0.355
Carcinoma	2 (1.4%)	0 (0%)	2 (0.8%)	0.503
Epidermoid carcinoma	0 (0%)	1 (0.8%)	1 (0.4%)	0.449
Leiomyosarcoma	1 (0.7%)	0 (0%)	1 (0.4)	>0.9
Ulceration				0.419
Not specified	11 (7.6%)	8 (6.8%)	19 (7.2%)	0.802
Ulcer-infiltrating Tumors	24 (16.6%)	27 (22.9%)	51 (19.4%)	0.197
Ulcer-vegetative tumor	51 (35.2%)	45 (38.1%)	96 (36.5%)	0.620
Mucoproducing Areas	24 (16.6%)	27 (22.88%)	51 (19.39%)	0.229
Angiolymphatic invasion	26 (17.9%)	30 (25.42%)	56 (21.29%)	0.197
Perioneural invasion	11 (7.58%)	5 (4.23%)	16 (6.08%)	0.221
Necrosis	24 (16.6%)	24 (20.33%)	48 (18.25%)	0.482
Unresectable tumor	4 (2.75%)	3 (2.54%)	7 (2.66%)	>0.9

Bold values indicate results considered relevant for interpretation (including statistically significant results and trends).

^a^ n (%); Mean (SD).

^b^ Pearson's Chi-squared test; Fisher's exact test; Wilcoxon rank sum test.

* Statistical significance (*p* < 0.05).

### 3.2 Rt-PCR SARS-CoV2 positive vs. negative

The impact of SARS-CoV-2 infection was analyzed in 87 surgical oncology patients, 70 of whom were SARS-CoV-2 negative and 17 SARS-CoV-2 positive (see Table 3). Compared to the pre-pandemic period, the positive patients exhibited a statistically significant difference in the outcomes of death, 30- day mortality, number of days of hospitalization, and need for intensive care.

**Table 3:** A comparative analysis of population clinical characteristics and histological variables in relation to SARS-CoV2 infection.

Variables	Negative Rt- PCR, *N* = 70^a^	Positive Rt- PCR, *N* = 17^a^	Total^a^	*p*-value^b^
Female	38 (54%)	5 (29%)	43 (49%)	0.066
Age				0.6
* *<60 years	18 (26%)	7 (41%)	25 (29%)	0.131
Death outcome	4 (5.7%)	6 (35%)	10 (11%)	0.003*
Days hospitalized	26.55 (17.00)	61.88 (81.64)	33.70 (41.46)	0.024*
Admission to intensive care unit	17 (24%)	9 (53%)	26 (30%)	0.021*
30-day mortality	4 (5.8%)	5 (29%)	9 (10%)	0.013*
Emergency surgery	21 (30%)	4 (24%)	25 (29%)	0.8
Palliative surgery	2 (2.9%)	2 (12%)	4 (4.6%)	0.2
ASA				0.2
1	2 (2.9%)	0 (0%)	2 (2.4%)	>0.9
2	40 (59%)	6 (40%)	46 (55%)	0.105
3	26 (38%)	8 (53%)	34 (41%)	0.452
4	0 (0%)	1 (6.7%)	1 (1.2%)	0.195

Bold values indicate results considered relevant for interpretation (including statistically significant results and trends).

^a^ *n* (%); Mean (SD).

^b^ Pearson's Chi-squared test; Fisher's exact test; Wilcoxon rank sum test.

* Statistical significance (*p* < 0.05).

### 3.3 Predictors

The pandemic period was evaluated as a possible predictor for intensive care unit admission, 30-day mortality, emergency surgery, palliative surgery, metastasis, advanced cancer, and T4 staging (see Table 4). The findings indicated that the pandemic period was a significant predictor of intensive care unit admissions, suggesting a reduction during the pandemic period. Furthermore, the pandemic period was significantly associated with emergency surgery, indicating an elevated likelihood of this outcome. Conversely, no statistically significant associations were observed between the pandemic period and 30- day mortality, the presence of metastases, advanced cancer, and T4 staging.

**Table 4:** Multivariate logistic regression model: association between the pandemic period and variables related to hospitalization and cancer staging.

Variables	OR (95% CI)	*p*-value
Admission to intensive care unit	0.50 (0.29–0.87)	**0** **.** **015** *
30-day mortality	2.0 (0.94–5.37)	0.15
Emergency surgery	2.0 (1.10–3.94)	**0** **.** **025** *
Palliative Surgery	0.84 (0.17–4.04)	0.83
Metastasis	0.985 (0.64–1.45)	0.93
Advanced cancer	1.16 (0.67–2.01)	0.57
T4	0.91 (0.52–1.51)	0.73

Bold values indicate results considered relevant for interpretation (including statistically significant results and trends).

* Statistical significance (*p* < 0.05).

In addition, the potential of Sars-CoV-2 infection as a predictor for admission to the intensive care unit, 30-day mortality, emergency surgery, and palliative surgery was evaluated (see Table 5).

**Table 5:** Multivariate logistic regression model: association between Sars-CoV-2 infection and variables related to hospitalization and cancer staging.

Variables	OR (95% CI)	*p*-value
Admission to intensive care unit	1.95 (0.54–6.89)	0.30
30-day mortality	3.97 (0.75–24.87)	0.10
Emergency surgery	0.70 (0.18–2.49)	0.61
Palliative Surgery	6.25 (0.59–80.34)	0.16

However, the investigation revealed no statistically significant association with any of the variables.

## 4 Discussion

The present study's findings demonstrated that the pandemic period was associated with increased emergency surgical procedures. Furthermore, the logistic regression analysis findings confirmed the pandemic period as a predictor of emergency surgeries. Concurrently, the pandemic period was associated with a reduced intensive care unit admission rate as the incidence of SARS-CoV-2 infection increased. This phenomenon can be attributed to the strategic reallocation of intensive care unit beds to patients infected with SARS-CoV-2.

The health system has encountered many challenges, resulting in delays in cancer treatment and the postponement and cancellation of various surgical procedures (4). An international prospective cohort study enrolling 20,006 adult patients (aged 18 years and over) from 466 hospitals across 61 countries, all diagnosed with 15 distinct types of cancer, underwent curative surgery during the pandemic. These patients were subsequently followed up until the time of their surgical procedure or until the cessation of follow-up. The study found that mild restrictions were associated with a non-operation rate of 0.6%, moderate lockdowns of 5.5%, and total lockdowns of 15-0%. The primary factors contributing to delays in the context of the study's focus on the SARS-CoV-2 included the multidisciplinary team's decision to postpone surgery due to the patient's risk during the pandemic period (72.8%), the transition to an alternative treatment modality (26.6%), and finally the patient's decision to forego surgery during the pandemic (23-0%) (4). According to recent findings (1), the postponement of cancer surgery by more than three and six months results in a 17% and 30% decline in survival rates for stage II and III gastrointestinal cancer patients, respectively. An international survey involving 76 centers found that 50% of curative cancer treatments were canceled during the pandemic (19).

An increase in the number of emergency procedures for surgical patients with colorectal cancer during the pandemic period compared to the pre-pandemic period was reported (3, 20, 21). The authors attributed this increase to cases of bowel obstruction or perforation. However, studies conducted by Tang et al. (22) and Rottoli et al. (23) reported no significant difference in the rate of emergency surgeries between the two periods. Several factors may explain this paradox: (1) Diagnostic delay and emergency presentation without pathological upstaging (24, 25). (2) Selective surgical access during the pandemic, where asymptomatic or early-stage cases were underdiagnosed due to reduced screening and outpatient visits. Consequently, many patients only sought treatment when experiencing severe symptoms, resulting in emergency interventions without prior histopathological confirmation of stage progression (25); (3) Possible underreporting of early-stage cases (25).

Notwithstanding the shift in the pattern of emergency surgeries and the necessity for intensive care, there was no difference in 30-day mortality and death outcomes during the pandemic period. As previously indicated, this is likely attributable to a reorganization of health services to address the demands of patients with SARS-CoV2, who exhibited a higher mortality rate compared to other patients. A meta-analysis revealed no difference in post-surgical mortality up to 90 days after surgery.

Merchant et al. (26) and Morris et al. (27) found no difference in the rate of T4 tumors during the pandemic, aligning with the results of our study. However, several authors have reported an increase in T4 tumors during the pandemic (3, 5, 15, 16, 21, 23). The present study observed a higher proportion of T3 cases during the pandemic period, suggesting a disease progression. A comprehensive review of the extant literature revealed no studies that reported an increase in T3 cases and a concomitant reduction in cases during the pandemic. Regarding ICU admissions, heightened preoperative risk assessments, improved perioperative protocols, and prioritizing stable oncologic patients for surgery during pandemic peaks may have helped maintain ICU admission rates despite increased comorbidities (28–30).

A meta-analysis (16) was conducted to compare tumor stages between 488 patients who underwent surgery before the emergence of the pandemic and 674 patients who underwent surgery during the pandemic. Contrary to the present study's findings, the meta-analysis revealed that the rate of T4 cases was significantly higher during the pandemic. The novel coronavirus (SARS-CoV-2) has been demonstrated to be an additional risk factor for oncological patients, increasing the likelihood of adverse outcomes. Patients with confirmed cases of SARS-CoV-2 have elevated rates of mortality, both in the immediate term and following 30 days, in addition to a significant duration of hospitalizations and intensive care unit admissions. It is noteworthy that advanced cancer was not associated with the pandemic period. However, a study (2) employing the same criteria for advanced cancer as the present study found a significant increase in this outcome during the pandemic period.

De Pelsemaeker et al. (31) illustrated a dramatic reduction in colon biopsy during the early months of the pandemic. Several European studies reported significant decreases in cancer screening procedures, including those for colorectal cancer. These delays have been linked to fewer early-stage diagnoses (32), and it has been projected that they will cause stage migration and increased mortality in colorectal cancer patients if they extend beyond six to twelve months (33). Meanwhile, studies have reported an increase in patients presenting with colorectal cancer complications, such as bowel obstruction, which often require emergency interventions. As observed during the pandemic, these late presentations were associated with more advanced disease stages and poorer prognostic indicators (34). Behavioral factors also played a pivotal role. Many individuals delayed seeking care until they experienced alarming symptoms, which reduced opportunities for early cancer diagnosis and increased the burden on emergency services when advanced-stage presentations surged (1, 35).

Moreover, oncologic care was impacted in terms of both diagnosis and treatment delivery. Multiple international studies reported widespread disruption in surgical scheduling and deferral of chemotherapy protocols (36). Disruptions to cancer care during the pandemic disproportionately affected vulnerable populations, especially racial and ethnic minorities who had limited access to telemedicine and digital health platforms (37).

The international scenario strongly resonates with the Brazilian experience. Kanno et al. (2023) analyzed emergency colorectal cancer surgeries in Brazil during the pandemic and found that most patients were treated only after severe complications, such as intestinal obstruction or perforation. They found that the proportion of stage IV tumors increased and that patients exhibited higher rates of vascular, lymphatic, and perineural invasion. These findings reflect the impact of delayed diagnosis and limited access to early treatment within the public health system (34). Studies in Brazil reported a reduction of over 45% in colorectal cancer (CRC) diagnostic procedures during the pandemic, particularly in regions with limited healthcare infrastructure (38).

In Brazil, the increase in advanced CRC during the pandemic likely led to a higher demand for emergency surgical interventions (38). National data also highlight structural challenges within the Brazilian healthcare system, including regional disparities in diagnosis rates and access to healthcare.

### 4.1 Limitations

The study has limitations. Its retrospective cohort design introduces the possibility of selection bias due to data collection from patients admitted to a single referral hospital. Consequently, the external validity of the findings might be constrained, precluding extrapolation to other populations or treatment contexts. Additionally, our study did not evaluate screening practices, presence of relapse, or detailed postoperative complication types.

The quality of the results is contingent upon the accuracy and completeness of institutional electronic records. Errors or omissions in these records can compromise the validity of the results.

## 5 Conclusions

Before the advent of the novel Coronavirus disease 2019 (COVID-19) pandemic, cancer was already a global health concern, primarily due to the challenges associated with diagnosis and the presence of advanced tumors even for pathologies that are amenable to screening tests, such as colonoscopy and mammography, among others. Beyond the challenges of delayed diagnosis, which complicates treatment and increases costs while reducing the likelihood of a cure, there is a growing concern of an exponential increase in the incidence of cancer over the next two decades. Consequently, health professionals were already concerned, and our study demonstrates the need to investigate the current scenario of health systems, especially for cancer patients. The pandemic has substantially impacted health, underscoring the need for comprehensive research to assess its impact, formulate countermeasures, and improve health at the regional, national, and global levels.

Our study demonstrates the importance of continuous treatment in cancer patients. Further efforts should be continued in multicenter studies to draw further conclusions. The decision to maintain surgical procedures for cancer patients, despite the incidence of SARS-CoV-2 infection during the pandemic and the meticulous preparation measures in place, did not result in any alteration in mortality rates for cancer patients.

## Data Availability

The data will be made available by the authors upon reasonable request.
